# Structure-Odor Relationships of α­Santalol Derivatives with Modified Side Chains

**DOI:** 10.3390/molecules17022259

**Published:** 2012-02-22

**Authors:** Toshio Hasegawa, Hiroaki Izumi, Yuji Tajima, Hideo Yamada

**Affiliations:** 1 Department of Chemistry, Graduate School of Science and Engineering, Saitama University, Saitama 338-8570, Japan; 2 Yamada-matsu Co., Ltd., Kyoto 602-8014, Japan

**Keywords:** α-santalol, side chain, * Z*-isomer, *E*-isomer, odor

## Abstract

(*Z*)-α-Santalol, which has a unique woody odor, is a main constituent of sandalwood essential oil. We investigated the structure-odor relationship of (*Z*)-α-santalol and its derivatives, focusing on the relationship between the structure of the side chain and the odor of the compounds. Various α-santalol derivatives (aldehydes, formates, and acetates) were synthesized from (*Z*)- and (*E*)-α-santalol, which were prepared from (+)-3-bromocamphor through modifications of a reported synthetic route. The *Z*- and *E*-isomers of α-santalols have different double-bond configurations in the side chain. Analogues with saturated side chains were also prepared from the corresponding α-santalols, and the odors of the all the prepared compounds were evaluated. We found that the odors of the *Z*-isomers (woody) were similar to those of the corresponding saturated compounds, but clearly different from the odors of the corresponding *E*-isomers (odorless, fresh, or fatty). These results indicate that the relative configuration of the side chain with respect to the santalane frame plays an important role in the odor of α-santalol. *E*-configuration in the side chain eliminates the woody odor character of α-santalol and its examined derivatives, whereas the *Z*-configuration or saturation of the carbon side chain does not.

## 1. Introduction

Sandalwood (*Santalum album* L.) is a valuable and expensive material because of the difficulty involved in growing sandalwood trees, from which the essential oil and incense are obtained. Although sandalwood trees are found in Malaysia and Australia, sandalwood trees of the highest quality for incense and perfume are only found in India (especially East India). The composition of sandalwood essential oils has been thoroughly investigated [1], and more than 300 constituents have been identified. The main constituents are (*Z*)-α-santalol (**1**) and (*Z*)-β-santalol. These compounds have distinctive woody odors. The structure-odor relationships of sandalwood odorants are an interesting topic in fragrance chemistry [2,3,4]. Furthermore, the structure-odor relationships of (*Z*)-β-santalol and its related compounds have been investigated in detail [5,6,7,8]. Previously, we reported that the odor of sandalwood chips is formed by a combination of santalols and their aldehyde and formate derivatives [9].

**Figure 1:**
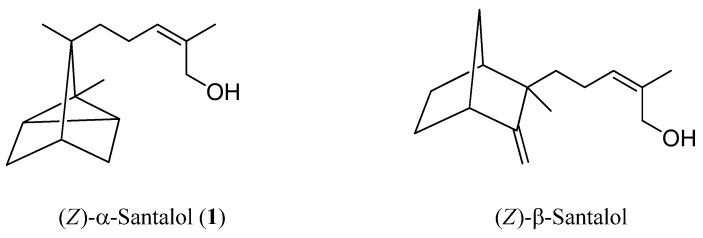
Main constituents of sandalwood.

We have evaluated the odors of santalyl aldehyde derivatives **4** and **5**, which were isolated from hexane extract [9]. The odor of *Z*-isomer **4** was sandalwood-like and found to be different from that of *E*-isomer **5** (slightly fatty). A similar result has been reported previously [10]. The *Z*-isomer of α-santalol has sandalwood odor, but the *E*-isomer does not. The odor of santalols depends on their *E-*/*Z-*configuration. In addition, Fanta *et al*. reported that the odors of dihydro-α-santalol and dihydro-β-santalol have sandalwood character [11]. In the present study, we investigated the relationship between the structure and odor of (*Z*)-α-santalol (**1**) (Figure 1) and its derivatives bearing modified side chains. To examine whether *E*-/*Z*-configuration alone or other structural elements of the aliphatic side chain of α-santalol and its derivative have an influence on the woody odor note, we focus our attention on the similarities and differences in odor between molecules with different side chain geometries (*Z*-isomer, *E*-isomer, and saturated side chain) and functional groups on the side chain.

## 2. Results and Discussion

### 2.1. Synthesis of α-Santalols

**Scheme 1:**
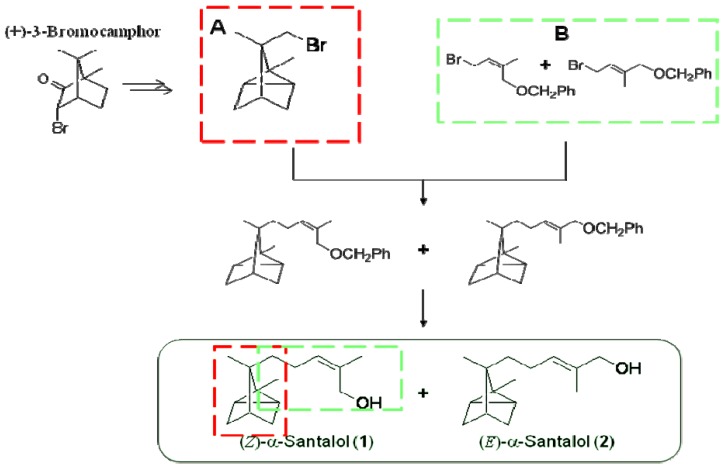
Synthetic route to *Z*-isomer (**1**) and *E*-isomer (**2**) of α-santalol.

There have been several reports on synthetic routes to (*Z*)-α-santalol (**1**). The first total synthesis was achieved by Corey *et al*. [12]. For the present study, both the *E*- and *Z*-isomers were synthesized from (+)-3-bromocamphor via a modified version of the route reported by Sato and co-workers [13]. In this route (Scheme 1), the two parts of α-santalol—the bicyclic frame (**A**) and the side chain (**B**)—were synthesized separately. The obtained parts were combined to give two α-santalol benzyl ethers with *Z*- and *E*-configurations.

Next, the synthesis from parts **A** and **B** to the targets (*Z*)-α-santalol (**1**) and (*E*)-α-santalol (**2**) is described. We first attempted to prepare α-santalol benzyl ethers by the Grignard reaction. However, only a coupling product was obtained, and the α-santalols could not be prepared. In a second attempt, a lithium reagent prepared from (−)-8-bromotricyclene (part **A**) was reacted with an allyl bromide (part **B**) to give the mixture of α-santalol benzyl ethers. The obtained ethers were converted to an *E/Z*-mixture of α-santalols. The resulting *E*/*Z*-mixture was separated by preparative thin layer chromatography (PTLC), which afforded the pure *Z*- and *E*-isomers.

**Figure 2:**
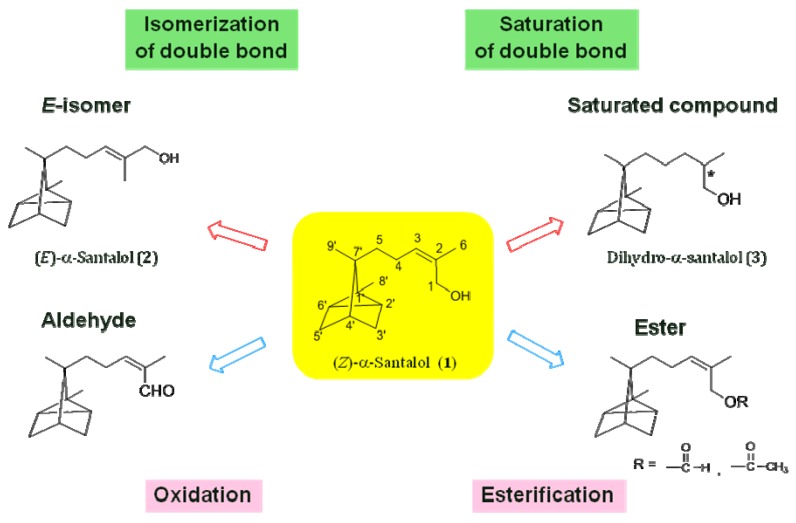
Structure–odor relationships of α-santalols considering the double bond and functional group of the side chain.

**Figure 3:**
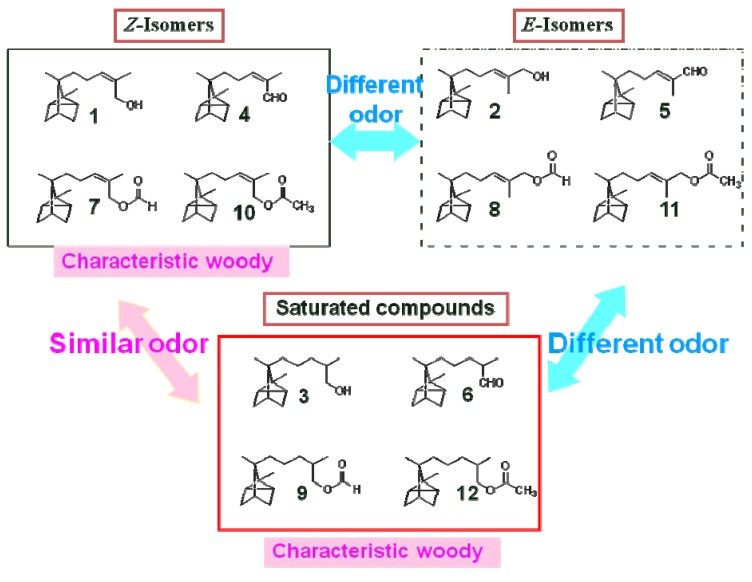
Odor comparison of α-santalol derivatives with four different functional groups.

We investigated the structure-odor relationships of α-santalol derivatives with different side-chain moieties by using the pure *Z*-isomer (**1**) and *E*-isomer (**2**) of α-santalol. We specifically looked at two structural aspects: the double bond and functional group of the side chain in α-santalol. As shown in Figure 2, the first structural aspect that we examined was the double bond in the α-santalol side chain. Specifically, we looked at the geometrical configuration (*Z*-isomer *vs. E*-isomer) and the presence or absence of the double bond (*Z*-isomer *vs.* saturated analogue). Figure 3 also shows the second structural aspect that we examined in relation to odor: the functional group of the side chain in α-santalol. We compared the odors of compounds bearing a hydroxy, formyloxy, or acetoxy group.

### 2.2. Synthesis and Odor Evaluation of a-Santalol Derivatives with Different Functional Groups in the Side Chain

**Scheme 2:**
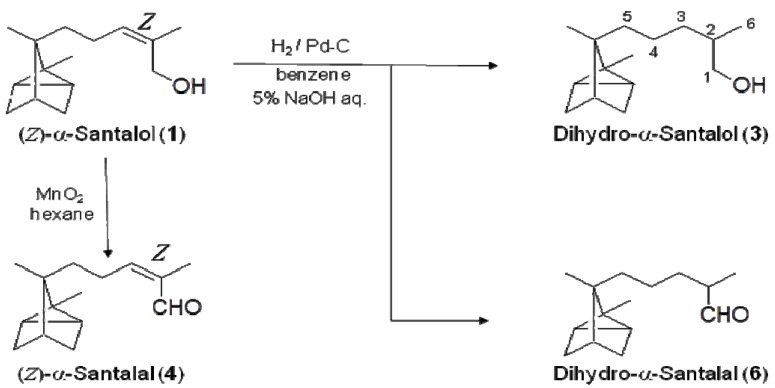
Synthesis of dihydro-α-santalols and aldehyde derivatives.

The syntheses of the aldehyde and saturated compounds are shown in Scheme 2. Dihydro-α-santalol (**3**) was obtained by hydrogenation of **1**. This reaction also produced dihydro-α-santalal (**6**), which is the aldehyde analogue of dihydro-α-santalol (**3**).

^13^C-NMR spectroscopy revealed that the dihydro-compounds were a 1:1 mixture of epimers due to the chirality of the C-2 carbon. The *Z*-isomer (**4**) and *E*-isomer (**5**) of α-santalal were synthesized by oxidation of (*Z*)- and (*E*)-α-santalols, respectively. We compared the odors of these compounds and evaluated the similarity of odors between the isomers. The odors of saturated compounds **3** and **6** were similar to those of *Z*-isomers **1** and **4**. In contrast, the odors of the *Z*-isomers were different from the odors of *E*-isomers **2** and **5** (Figure 3).

The formates and acetates of α-santalol were prepared from the corresponding α-santalols (for details, see the Experimental section). We found the characteristic aroma features of these compounds by sensory evaluation and GC-O (retention index (RI) for each compound: **1**: 2,305; **2**: 2,342; **3**: 2,250; **7**: 2,184; **8**: 2,239; **9**: 2,153; **10**: 2,205; **11**: 2,276; **12**: 2,171). We were not able to evaluate the odors of aldehyde derivatives **4**, **5**, and **6** by GC-O because of the thermal instability of these compounds. The compounds gradually decomposed at room temperature.

The characteristic sandalwood-like woody odors of the *Z*-isomers of α-santalol derivatives (**1**: woody; **4**: sweet, woody; **7**: green, woody; **10**: fatty, woody) were similar to those of the saturated compounds (**3**: weak sweet, woody; **6**: fresh green, woody; **9**: floral, woody; **12**: fatty, woody), but the odors of the *E*-isomers (**2**: odorless; **5**: slightly fatty; **8**: slightly fresh; **11**: odorless) were different from those of the *Z*-isomers and the saturated compounds.

The side chain in the *Z*- and *E*-isomer of α-santalol is rigid; in contrast, the side chain in the saturated compounds is flexible and can adopt various conformations due to the free rotation about the single bond. The most stable conformer is presumed to be the antiperiplanar form, which is similar to the structure of the *E*-isomer. The odors of the saturated compounds are similar to those of the *Z*-isomers, which is similar to the unfavorable synperiplanar form of the saturated compounds. These results suggest that the odor receptor requires a structure like the *Z*-configuration of these odor compounds in order to produce a sandalwood-like odor.

## 3. Experimental

### 3.1. General

All commercially obtained chemicals were used as received. (+)-3-Bromocamphor was purchased from Wako Pure Chemical Industries, Ltd., Japan. The solvents used in synthesis were distilled by an appropriate purification procedure. The structures of the reported compounds were determined by 1D and 2D NMR (H,H-COSY; C,H-COSY; HMBC) studies through comparison with the reported ^1^H-NMR and ^13^C-NMR spectroscopic data on α-santalol (**1**) [6]. Chemical shifts are expressed in ppm using TMS as an internal standard. NMR spectra were recorded on a AVANCE500 spectrometer (Bruker, Germany). Low-resolution mass spectrometry (MS) was performed on a JMS-700 AM spectrometer (JEOL, Japan) using electron impact (EI) ionization (70 eV), and high-resolution mass spectrometry (HRMS) was performed on a JMS-T100GCV spectrometer (JEOL, Japan) using field ionization mode. Silica Gel 60 GF254 was used for TLC. Silica Gel 60 PF254 was used for PTLC. Preparative high performance liquid chromatography (HPLC) was performed on an LC-9101 system (Japan Analytical Industry, Tokyo, Japan) equipped with a UV detector (210 nm) and 5SIL 10E column (neutral silica gel, Shodex, Tokyo, Japan; 250 mm × 10 mm i.d.; particle size: 5 μm). Gas chromatograph olfactometry (GC-O) analysis was performed on a GC-353 gas chromatograph (GL Sciences, Japan) equipped with an InertCap Pure-WAX capillary column (30 m × 0.25 mm i.d.; film thickness: 0.25 μm) and an OP 275 unit. The carrier gas was helium at a flow rate of 1 mL/min. The injections were performed in splitless mode at 250 °C. One microliter of oil solution in hexane (HPLC grade) was injected. The following temperature program was used: 40 °C for 5 min, followed by an increase to 250 °C at 6 °C/min. RIs were calculated using a series of *n*-alkanes (C_16_, C_17_, C_18_, and C_20_).

### 3.2. Sensory Evaluation

Sensory evaluation of the synthesized compounds without solvent was performed by an expert panel consisting of four members of Yamada-matsu Co., Ltd. The similarities and differences in odor character of the compounds were evaluated. The sensory evaluation was also performed by a non-expert panel consisting of untrained participants. The sensory evaluation results were consistent between the expert and non-expert panels.

### 3.2. Synthesis of a-Santalyl Benzyl Ethers and Separation of Their (Z)- and (E)-Isomers

A solution of *t*-BuLi in pentane (1.60 mol/L, 20.0 mL, 32.0 mmol) was cooled to −78 °C in a dry ice-methanol bath under a nitrogen atmosphere. An absolute ether solution (40 mL) of (−)-8-bromotricyclene (3.03 g, 14.1 mmol) was added dropwise to the solution over 1.5 h. After 10 min, the reaction mixture was warmed to 25 °C and stirred for 2 h. The reaction mixture was again cooled to −78 °C, and ether solution of 1-benzyloxy-4-bromo-2-methyl-2-butene (2.02 g, 7.92 mmol; *E*/*Z*-isomer = 62:38) was added dropwise to the solution over 1 h. The reaction mixture was warmed to 25 °C and held at that temperature. After 12 h the completion of the reaction was observed by TLC [(SiO_2_, hexane-AcOEt (95:5)]. Saturated ammonium chloride solution (20 mL) and ether (150 mL) were added to the reaction mixture. The ether solution was washed with saturated ammonium chloride solution (20 mL × 2) and saturated sodium chloride solution (20 mL × 2). The solution was then dried over anhydrous magnesium sulfate. Removal of the solvent under reduced pressure gave an oil. The oil was purified by bulb-to-bulb distillation (130–140 °C at 0.06 Torr) to afford α-santalyl benzyl ethers (1.03 g, 42%; *E*/*Z*-isomer = 62:38) as a colorless oil. The *E/Z*-mixture was separated by HPLC and isolated as colorless oils.

*(Z)-Benzyl ethers*. Colorless liquid; ^1^H-NMR (500 MHz, CDCl_3_) δ 0.82−0.90 (m, 3H, H-2′,6′), 0.86 (s, 3H, H-9′), 0.98 (s, 3H, H-8′), 1.03−1.08 (m, 1H, H-4′), 1.11−1.16 (m, 2H, H-3′,5′), 1.25−1.30 (m, 5H, H-5), 1.51−1.60 (m, 2H, H-3′,5′), 1.79 (s, 3H, H-6), 1.95−2.00 (m, 2H, H-4), 4.01 (s, 2H, H-1), 4.45 (s, 2H, H-1′′), 5.39 (t, 1H, H-3, *J* = 7.0 Hz), 7.33−7.36 (m, 5H, H-2′′−5′′); ^13^C-NMR (125 MHz, CDCl_3_) δ 10.65 (C-8′), 17.51 (C-9′), 19.49 (C-2′), 19.52 (C-6′), 21.25 (C-6), 22.93 (C-4), 27.37 (C-1′), 31.12 (C-5′), 31.51 (C-3′), 35.00 (C-5), 38.17 (C-4′), 45.87 (C-7′), 61.60 (C-1), 129.53 (C-3), 133.67 (C-2).

*(E)-Isomer of the benzyl ethers*. Colorless liquid; ^1^H-NMR (500 MHz, CDCl_3_) δ 0.75−0.90 (m, 2H, H-2′,6′), 0.87 (s, 3H, H-9′), 1.00 (s, 3H, H-8′), 1.03−1.08 (m, 1H, H-4′), 1.11−1.20 m, (2H, H-3′,5′), 1.24−1.27 (m, 2H, H-5), 1.51−1.60 (m, 2H, H-3′,5′), 1.68 (s, 3H, H-6), 1.94−2.03 (m, 2H, H-4), 3.90 (s, 2H, H-1), 4.45 (s, 2H, H-1′′), 5.43 (t, 1H, H-3, *J* = 7.0 Hz), 7.34−7.36 (m, 5H, H-2′′−5′′); ^13^C-NMR (125 MHz, CDCl_3_) δ 10.65 (C-8′), 13.53 (C-6), 17.51 (C-9′), 19.51 (C-2′), 19.56 (C-6′), 22.91 (C-4), 27.41 (C-1′), 31.01 (C-5′), 31.52 (C-3′), 34.22 (C-5), 38.19 (C-4′), 45.87 (C-7′), 69.13 (C-1), 1297.34 (C-3), 134.14 (C-2).

### 3.3. Synthesis of α-Santalol from α-Santalyl Benzyl Ether and Separation of Its (Z)-Isomer *(**1**)* and (E)-Isomer *(**2**)*

A round-bottom flask was cooled to −78 °C in a dry ice-methanol bath under nitrogen atmosphere. Ethylamine (dried with potassium hydroxide; 19 mL) was added to the flask. Flakes of lithium were added to the ethylamine, and after 10 min, the solution became deep blue. After the solution was stirred for 40 min, a hexane solution (8 mL) of α-santalyl benzyl ether (832 mg, 2.68 mmol) (*E*/*Z*-isomer = 62:38) was added dropwise over 30 min. The progress of the reaction was monitored by TLC [(SiO_2_, hexane-AcOEt (10:3)]. Ammonium chloride solution was added to the reaction mixture until the blue color disappeared. Methanol (10 mL) was added, and ethylamine was removed under reduced pressure from the reaction mixture at room temperature. The solution was extracted with ether (50 mL × 3). The obtained solution was washed with saturated sodium chloride solution (20 mL × 3). The organic solution was dried over anhydrous magnesium chloride, and removal of the solvent gave a crude mixture, which was purified by column chromatography [SiO_2_, hexane-AcOEt (10:3)] to give the *E/Z*-mixture of α-santalol (331 mg, 56%; *E*/*Z*-isomer = 62:38) as a colorless liquid. Repeated purification by PTCL [SiO_2_; first, hexane-AcOEt (70:30); second, hexane-isopropanol (90:10)] afforded (*Z*)-α-santalol (**1**; 88.3 mg) and (*E*)-α-santalol (**1**; 75.1 mg) as pure colorless oils. 

*(Z)-α-Santalol* (**1**). Colorless liquid; ^1^H-NMR (500 MHz, CDCl_3_) δ 0.82−0.85 (m, 2H, H-2′,6′), 0.83 (s, 3H, H-9′), 0.99 (s, 3H, H-8′), 1.03−1.08 (m, 2H, H-3′,5′), 1.11−1.17 (m, 1H, H-5), 1.20−1.27 (m, 1H, H-5), 1.55−1.62 (m, 3H, H-3′,4′,5′), 1.79 (s, 3H, H-6), 1.94−2.00 (m, 2H, H-4), 4.14 (s, 2H, H-1), 5.31 (t, 1H, *J* = 7.5 Hz, H-3); ^13^C-NMR (125 MHz, CDCl_3_) δ 10.65 (C-8′), 17.51 (C-9′), 19.49 (C-2′), 19.52 (C-6′), 21.25 (C-6), 22.93 (C-4), 27.37 (C-1′), 31.12 (C-5′), 31.51 (C-3′), 35.00 (C-5), 38.17 (C-4′), 45.87 (C-7′), 61.60 (C-1), 129.53 (C-3), 133.67 (C-2).

*(E)-α-Santalol* (**2**). Colorless liquid; ^1^H-NMR (500 MHz, CDCl_3_) δ 0.84−0.90 (m, 2H, H-2′,6′), 0.87 (s, 3H, H-9′), 1.00 (s, 3H, H-8′), 1.02−1.08 (m, 2H, H-3′,5′), 1.12−1.20 (m, 1H, H-5), 1.22−1.35 (m, 1H, H-5), 1.57−1.63 (m, 3H, H-3′,4′,5′), 1.67 (s, 3H, H-6), 1.92−2.02 (m, 2H, H-4), 4.00 (s, 2H, H-1), 5.41 (t, 1H, *J* = 7.0 Hz, H-3); ^13^C-NMR (125 MHz, CDCl_3_) δ 10.65 (C-8′), 13.53 (C-6), 17.51 (C-9′), 19.51 (C-2′), 19.56 (C-6′), 22.91 (C-4), 27.41 (C-1′), 31.01 (C-5′), 31.52 (C-3′), 34.22 (C-5), 38.19 (C-4′), 45.87 (C-7′), 69.13 (C-1), 127.34 (C-3), 134.14 (C-2).

### 3.4. Synthesis of Dihydro-α-Santalol *(**3**)* and Dihydro-α-Santalal (6)

To a flask that had been purged with nitrogen, 5% Pd-C (22.5 mg) was added. The *E/Z*-mixture of α-santalol (*E*/*Z*-isomer = 62:38; in 1 mL of benzene), benzene (2 mL), and 5% aqueous sodium hydroxide solution (1 mL) were added to the flask. Hydrogen was introduced to the flask, and the solution was stirred under hydrogen atmosphere for 8 h at room temperature. The progress of the reaction was monitored by TLC (SiO_2_, CHCl_3_). Pd-C was filtered from the solution, and removal of the solvent gave a crude oil. The mixture was purified by PTLC (SiO_2_, CHCl_3_) to afford dihydro-α-santalol (**3**; 5.7 mg, 25%) and dihydro-α-santalal (**6**; 4.7 mg, 21%).

*Dihydro-α-santalol* (**3**). Colorless liquid; ^1^H-NMR (500 MHz, CDCl_3_) δ 0.79−0.88 (m, 2H, H-2′,6′), 0.82 (s, 3H, H-9′), 0.92 (d, 3H, *J* = 6.6 Hz, H-6), 0.99 (s, 3H, H-8′), 1.01−1.06 (m, 2H, H-3′,5′), 1.10–1.38 (m, 6H, H-3,4,5), 1.54−1.67 (m, 3H, H-3′,4′,5′), 1.68−1.81 (m, 1H, H-2), 3.42 (dd, 1H, *J* = 10.5, 6.5 Hz, H-1), 3.51 (dd, 1H, *J* = 10.5, 6.5 Hz, H-1); ^13^C-NMR (125 MHz, CDCl_3_) δ 10.65 (C-8′), 16.57 and 16.64 (C-6), 17.61 (C-9′), 19.52 (C-2′,6′), 21.81 and 21.85 (C-4), 27.37 (C-1′), 31.02 (C-5′), 31.48 (C-3′), 34.19 and 34.20 (C-3), 34.54 (C-5), 35.68 and 35.70 (C-2), 38.23 (C-4′), 45.84 (C-7′), 68.44 and 68.52 (C-1).

*Dihydro-α-santalal* (**6**). Colorless liquid; ^1^H-NMR (500 MHz, CDCl_3_) δ 0.79−0.88 (m, 2H, H-2′,6′), 0.86 (s, 3H, H-9′), 1.09 (d, 3H, *J* = 7.0 Hz, H-6), 1.12−1.43 (m, 10H), 1.50−1.62 (m, 3H), 1.65–1.75 (m, 1H), 2.30–2.38 (m, 1H, H-2), 9.62 (d, 1H, *J* = 3.5 Hz, H-1); ^13^C-NMR (125 MHz, CDCl_3_) δ 10.7 (C-8′), 13.35 and 13.41 (C-6), 17.6 (C-9′), 19.5 (C-2′, 6′), 21.94 and 21.96 (C-4), 27.4 (C-1′), 31.0 (C-5′ or C-3′), 31.5 (C-3′ or C-5′), 31.58 and 31.63 (C-3), 34.4 (C-5), 38.2 (C-4′), 45.8 (C-7′), 46.31 and 46.37 (C-2), 205.4 (C-1) MS (*m*/*z*, %) 220 (M^+^, 7), 187 (31), 171 (21), 153 (49), 131 (97), 121 (91), 93 (100), 87 (98), 79 (25), 77 (16). HRMS (*m*/*z*): [M]^+^ calcd for C_15_H_24_O, 220.1827; found, 220.1822.

### 3.5. Synthesis of Aldehyde Derivatives of α-Santalol

(*Z*)-α-Santalol (**1**; 8.0 mg, 0.036 mmol) was dissolved in hexane (0.5 mL). Manganese dioxide (70.8 mg) was added to the solution and stirred for 24 h. The progress of the reaction was monitored by TLC (SiO_2_, CHCl_3_). Hexane (5 mL) was added to the reaction mixture, and the solid was filtered and washed with chloroform (5 mL). Removal of organic solvent from the obtained solution gave (Z)-α-santalal (**4**; 7.1 mg, 96%) as a colorless oil. Through a similar procedure, (*E*)-α-santalal (**5**; 8.0 mg, 80%) was synthesized from (*E*)-α-santalol (**2**; 10.0 mg, 0.045 mmol).

*(Z)-α-Santalal* (**4**). Colorless liquid; ^1^H-NMR (500 MHz, CDCl_3_) δ 0.80−0.92 (m, 2H, H-2′,6′), 0.86 (s, 3H, H-9′), 1.01 (s, 3H, H-8′), 0.99−1.12 (m, 3H, H-3′,4′,5′), 1.24−1.42 (m, 2H, H-5), 1.57−1.65 (m, 2H, H-3′,5′), 1.77 (s, 3H, H-6), 2.44−2.55 (m, 2H, H-4), 6.54 (t, 1H, H-3, *J* = 8.4 Hz), 10.16 (s, 1H, H-1); ^13^C-NMR (125 MHz, CDCl_3_) δ 10.65 (C-8′), 17.51 (C-9′), 19.49 (C-2′), 19.52 (C-6′), 21.25 (C-6), 22.93 (C-4), 27.37 (C-1′), 31.12 (C-5′), 31.51 (C-3′), 35.00 (C-5), 38.17 (C-4′), 45.87 (C-7′), 61.60 (C-1), 129.53 (C-3), 133.67 (C-2). 

*(E)-α-Santalal* (**5**). Colorless liquid; ^1^H-NMR (500 MHz, CDCl_3_) δ 0.80−0.92 (m, 2H, H-2′,6′), 0.86 (s, 3H, H-9′), 1.00 (s, 3H, H-8′), 1.04−1.16 (m, 3H, H-3′,4′,5′), 1.25−1.44 m, (2H, H-5), 1.57−1.63 (m, 2H, H-3′,5′), 1.75 (s, 3H, H-6), 2.23−2.35 (m, 2H, H-4), 6.50 (t, 1H, H-3, *J* = 7.5 Hz), 9.39 (s, 1H, H-1); ^13^C-NMR (125 MHz, CDCl_3_) δ 10.65 (C-8′), 13.53 (C-6), 17.51 (C-9′), 19.51 (C-2′), 19.56 (C-6′), 22.91 (C-4), 27.41 (C-1′), 31.01 (C-5′), 31.52 (C-3′), 34.22 (C-5), 38.19 (C-4′), 45.87 (C-7′), 69.13 (C-1), 1297.34 (C-3), 134.14 (C-2).

### 3.6. Synthesis of Formates of α-Santalol

(*Z*)-α-Santalol (**1**; 6.2 mg, 0.028 mmol) was dissolved in absolute benzene (1.0 mL). Formic acid (30 μL, 0.78 mmol) and anhydrous magnesium sulfate (87 mg) were added to the solution and stirred overnight. The reaction was monitored by TLC (SiO_2_, CHCl_3_). A large amount of **1** was not consumed. Formic acid (30 μL, 0.78 mmol) and anhydrous magnesium sulfate (142 mg) were also added to the solution. The solution was stirred overnight, and the progress of the reaction was monitored by TLC. The reaction mixture was extracted with benzene (5 mL × 4). The solution was washed with saturated sodium hydrogen carbonate solution (2 mL × 2) and saturated sodium chloride solution (2 mL × 2). The obtained organic solution was dried over anhydrous magnesium sulfate. Removal of the solvent from the reaction mixture gave (*Z*)-α-santalyl formate (**7**; 6.9 mg, 99%) as a colorless oil. In a similar procedure, the reaction of (*E*)-α-santalol (**2**; 9.2 mg, 0.042 mmol) and dihydro-α-santalol (**3**; 6.5 mg, 0.029 mmol) gave (*E*)-α-santalyl formate (**8**; 10.2 mg, 98%) and dihydro-α-santalyl formate (**9**; 7.1 mg, 97%), respectively.

*(Z)-α-Santalyl formate* (**7**). Colorless liquid; ^1^H-NMR (500 MHz, CDCl_3_) δ 0.82−0.88 (m, 2H, H-2′,6′), 0.83 (s, 3H, H-9′), 0.99 (s 3H, H-8′), 1.04−1.08 (m, 2H, H-3′,5′), 1.12−1.18 (m, 1H, H-5), 1.23−1.33 (m, 1H, H-5), 1.55−1.62 (m, 3H, H-3′,4′,5′), 1.78 (s, 3H, H-6), 1.96−2.05 (m, 2H, H-4), 4.69 (s, 2H, H-1), 5.43 (t, 1H, *J* = 7.5 Hz, H-3), 8.11 (s, 1H, -CHO); ^13^C-NMR (125 MHz, CDCl_3_) δ 10.64 (C-8′), 17.49 (C-9′), 19.49 (C-2′), 19.53 (C-6′), 21.41 (C-6), 23.18 (C-4), 29.39 (C-1′), 30.99 (C-5′), 31.51 (C-3′), 34.62 (C-5), 38.16 (C-4′), 45.87 (C-7′), 62.55 (C-1), 128.47 (C-3), 132.53 (C-2), 161.12 (-CHO).

*(E)-α-Santalyl formate* (**8**). Colorless liquid; ^1^H-NMR (500 MHz, CDCl_3_) δ 0.83−0.89 (m, 2H, H-2′,6′), 0.84 (s, 3H, H-9′), 1.00 (s, 3H, H-8′), 1.02−1.08 (m, 2H, H-3′,5′), 1.12−1.19 (m, 1H, H-5), 1.23−1.29 (m, 1H, H-5), 1.56−1.63 (m, 3H, H-3′,4′,5′), 1.76 (s, 3H, H-6), 1.94−2.02 (m, 2H, H-4), 4.55 (s, 2H, H-1), 5.51 (t, 1H, H-3, *J* = 7.3 Hz), 8.10 (s, 1H, -CHO); ^13^C-NMR (125 MHz, CDCl_3_) δ 10.64 (C-8′), 13.75 (C-6), 17.49 (C-9′), 19.50 (C-6′), 19.56 (C-2′), 223.12 (C-4), 27.40 (C-1′), 31.01 (C-3′), 31.52 (C-5′), 33.91 (C-5), 38.19 (C-4′), 45.86 (C-7′), 69.91 (C-1), 128.79 (C-3), 131.66 (C-2), 161.02 (-CHO); MS (*m*/*z*, %) 248 (M^+^, 1), 202 (36), 187 (20), 135 (10), 121 (35), 107 (40), 93 (100), 91 (73), 79 (50), 77 (49). HRMS (*m*/*z*): [M]^+^ calcd for C_16_H_24_O_2_, 240.1776; found, 248.1779.

*Dihydro-α-santalyl formate* (**9**). Colorless liquid; ^1^H-NMR (500 MHz, CDCl_3_) δ 0.80−0.89 (m, 2H, H-2′,6′), 0.83 (s, 3H, H-8′), 0.95 (d, 3H, *J* = 6.5 Hz, H-6), 0.99 (s, 3H, H-8′), 1.01−1.06 m, (2H, H-3′,5′), 1.11−1.37 (m, 6H, H-3,4,5), 1.53−1.67 (m, 3H, H-3′,4′,5′), 1.80−1.85 (m, 1H, H-2), 3.96 (dd, 1H, *J* = 10.8, 6.8 Hz, H-1), 4.06 (dd, 1H, *J* = 10.8, 5.8 Hz, H-1), 8.09 (s, 1H, -CHO); ^13^C-NMR (125 MHz, CDCl_3_) δ 10.68 (C-8′), 16.78 and 16.86 (C-6), 17.60 (C-9′), 19.52 (C-2′,6′), 21.67 and 21.69 (C-4), 27.38 (C-1′), 32.40 (C-3), 31.02 (C-5′), 31.48 (C-3′), 34.30 (C-5), 34.32 and 34.45 (C-2), 38.22 (C-4′), 45.83 (C-7′), 68.91 and 68.96 (C-1), 161.28 (-CHO); MS (*m*/*z*, %) 250 (M^+^, 42), 205 (6), 121 (100), 93 (86), 91 (25), 79 (22), 77 (14). HRMS (*m*/*z*): [M]^+^ calcd for C_16_H_26_O_2_, 250.1933; found, 250.1934.

### 3.7. Synthesis of Acetates of α-Santalol

(*Z*)-α-Santalol (**1**; 5.7 mg, 0.026 mmol), triethylamine (0.4 mL), anhydrous acetic acid (0.020 mL, 0.21 mmol), and dimethylaminopyridine (2.4 mg) were added to a flask and stirred for 12 h. The progress of the reaction was monitored by TLC (SiO_2_, chloroform). The reaction mixture was diluted with hexane (30 mL). The solvent was washed with 1 mol/L HCl (3 mL × 2), saturated sodium hydrogen carbonate solution (3 mL × 2), and saturated sodium chloride solution (3 mL × 2). The solution was dried over anhydrous magnesium sulfate. Removal of the organic solvent gave (*Z*)-santalyl acetate (**10**; 5.8 mg, 85%) as a colorless oil. The (*E*)-isomer (**11**) and dihydro derivative (**12**) were synthesized from (*E*)-α-santalol (**2**) and dihydro-α-santalol (**2**), respectively, through the same procedure; The acetate (6.4 mg, 79%) was synthesized from (*E*)-α-santalol (6.6 mg, 0.030 mol), and the acetate (2.3 mg, 96%) was synthesized and from dihydro-α-santalol (2.0 mg, 0.0091 mmol).

*(Z)-α-Santalyl acetate* (**10**). Colorless liquid; ^1^H-NMR (500 MHz, CDCl_3_) δ 0.82 (s, 3H, H-9′), 0.83−0.87 (m, 2H, H-2′,6′), 0.99 (s, 3H, H-8′), 1.03−1.07 (m, 2H, H-3′,5′), 1.12−1.18 (m, 1H, H-5), 1.21−1.28 (m, 1H, H-5), 1.55−1.62 (m, 3H, H-3′,4′,5′), 1.74 (s, 3H, H-6), 1.96−2.06 (m, 2H, H-4), 2.07 (s, 3H, COCH_3_), 4.59 (s, 2H, H-1), 5.41 (t, 1H, *J* = 7.5 Hz, H-3); ^13^C-NMR (125 MHz, CDCl_3_) δ 10.64 (C-8′), 17.49 (C-9′), 19.49 (C-2′), 19.53 (C-6′), 20.98 (COCH_3_), 21.47 (C-6), 23.13 (C-4), 27.38 (C-1′), 30.99 (C-5′), 31.50 (C-3′), 34.66 (C-5), 38.15 (C-4′), 45.86 (C-7′), 63.19 (C-1), 129.15 (C-3), 131.87 (C-2), 171.21 (-CO-); MS (*m*/*z*, %) 262 (M^+^, 1), 202 (58), 187 (23), 135 (19), 121 (88), 107 (44), 94 (100), 93 (97), 79 (36), 77(24). HRMS (*m*/*z*): [M]^+^ calcd for C_17_H_26_O_2_, 262.1933; found, 262.1937.

*(E)-α-Santalyl acetate* (**11**). Colorless liquid; ^1^H-NMR (500 MHz, CDCl_3_) δ 0.83−0.88 (m, 2H, H-2′,6′), 0.84 (s, 3H, H-9′), 1.00 (s, 3H, H-8′), 1.02−1.08 (m, 2H, H-3′,5′), 1.13−1.19 (m, 1H, H-5), 1.23−1.29 (m, 1H, H-5), 1.57−1.62 (m, 3H, H-3′,4′,5′), 1.67 (s, 3H, H-6), 1.94−2.00 (m, 2H, H-4), 2.08 (s,3H, COCH_3_), 4.45 (s, 2H, H-1), 5.41 (t, 1H, *J* = 7.0 Hz, H-3); ^13^C-NMR (125 MHz, CDCl_3_) δ 10.64 (C-8′), 13.81 (C-6), 17.48 (C-9′), 19.50 (C-2′), 19.55 (C-6′), 21.04 (COCH_3_), 23.06 (C-4), 27.39 (C-1′), 31.00 (C-5′), 31.51 (C-3′), 33.97 (C-5), 38.17 (C-4′), 45.85 (C-7′), 70.40 (C-1), 129.39 (C-3), 130.73 (C-2), 171.06 (-CO-); MS (*m*/*z*, %) 262 (M^+^, 1), 202 (97), 187 (43), 135 (31), 121 (99), 107 (74), 94 (100), 93 (99), 79 (58), 77 (37). HRMS (*m*/*z*): [M]^+^ calcd for C_17_H_26_O_2_, 262.1933; found, 262.1937.

*Dihydro-α-santalyl acetate* (**12**). Colorless liquid; ^1^H-NMR (500 MHz, CDCl_3_) δ 0.79−0.88 (m, 2H, H-2′,6′), 0.86 (s, 3H, H-9′), 0.94 (d, 3H, *J* = 6.5 Hz, H-6), 0.98−1.45 (m, 12H), 1.52−1.67 (m, 3H), 2.06 (s, 3H, -COCH_3_), 3.84 (dd, 1H, *J* = 11.0, 6.5 Hz, H-1), 3.95 (dd, 1H, *J* = 11.0, 6.5 Hz, H-1); ^13^C-NMR (125 MHz, CDCl_3_) δ 10.69 (C-8′), 16.86 (C-6), 17.60 (C-9′), 19.51 (C-2′,6′), 20.99 (COCH_3_), 21.68 (C-4), 27.77 (C-1′), 31.01 (C-5′), 31.47 (C-3′), 32.53 and 32.42 (C-3), 33.43 (C-5), 34.46 (C-2), 38.21 (C-4′), 44.20 (C-7′), 69.53 and 69.63 (C-1), 171.34 (-OCO-); MS (*m*/*z*, %) 264 (M^+^, 34), 249 (2), 221 (3), 135 (11), 121 (100), 119 (18), 93 (72), 91 (18), 79 (16), 77 (14). HRMS (*m*/*z*): [M]^+^ calcd for C_17_H_28_O_2_, 260.2089; found, 260.2090.

## 4. Conclusions

We have synthesized several types of α-santalol derivatives that differed only in terms of their side-chain moiety. We found that the odors of the *Z*-isomers of α-santalol derivatives are similar to those of the corresponding saturated compounds, but clearly different from those of the *E*-isomers. These results indicate that the relative configuration of side-chain moiety with respect to the santalane frame plays an important role in creating the characteristic odor of these compounds.

## Supplementary Materials

Supplementary File 1
