# Hasegawa, T., *et al.* Structure-Odor Relationships of α­Santalol Derivatives with Modified Side Chains. *Molecules*, 2012, *17*, 2259-2270

**DOI:** 10.3390/molecules170911292

**Published:** 2012-09-21

**Authors:** Toshio Hasegawa, Hiroaki Izumi, Yuji Tajima, Hideo Yamada

**Affiliations:** 1 Department of Chemistry, Graduate School of Science and Engineering, Saitama University, Saitama 338-8570, Japan; 2 Yamada-matsu Co., Ltd., Kyoto 602-8014, Japan

The authors are sorry to report that some of the ^13^C-NMR data reported in their recently published paper [1] were incorrect. While this manuscript was in preparation and pending recording of some ^13^C-NMR spectra the data of α-santalol was used as a placeholder. Upon revising the manuscript according to the reviewers’ comments, they mistakenly thought that all spectral data had been replaced by the correct measured data. Consequently the authors wish to make at this time the following corrections to the paper:

Section *3.2. Synthesis of a-Santalyl Benzyl Ethers and Separation of Their (Z)- and (E)-Isomers*

(*Z*)*-Benzyl ether*. ^13^C-NMR (125 MHz, CDCl_3_) δ 10.65 (C-8′), 17.47 (C-9′), 19.49 (C-2′), 19.51 (C-6′), 21.69 (C-6), 23.03 (C-4), 27.36 (C-1′), 31.00 (C-5′), 31.50 (C-3′), 34.83 (C-5), 38.13 (C-4′), 45.84 (C-7′), 68.39 (C-1), 71.68 (C-7′′), 127.5 (C-4′′), 127.7 (C-3′′,5′′), 128.3 (C-2′′,6′′), 130.6 (C-3), 131.4 (C-2), 138.6 (C-1′′). 

(*E*)*-Isomer of the benzyl ether*. ^13^C-NMR (125 MHz, CDCl_3_) δ 10.67 (C-8′), 13.80 (C-6), 17.51 (C-9′), 19.52 (C-2′), 19.56 (C-6′), 23.02 (C-4), 27.40 (C-1′), 31.02 (C-5′), 31.52 (C-3′), 34.18 (C-5), 38.19 (C-4′), 45.88 (C-7′), 71.47 (C-7′′), 76.42 (C-1), 127.5 (C-3), 127.7 (C-4′′), 128.3 (C-3′′,5′′), 129.2 (C-2′′,6′′), 131.7 (C-2), 138.7 (C-1′′).

Section *3.5. Synthesis of α-Santalol Aldehyde Derivatives*

(*Z*)-*α-Santalal* (**4**). ^13^C-NMR (125 MHz, CDCl_3_) δ 10.51 (C-8′), 16.32 (C-6), 17.43 (C-9′), 19.35 (C-2′), 19.47 (C-6′), 22.26 (C-4), 27.31 (C-1′), 30.96 (C-5′), 31.45 (C-3′), 34.80 (C-5), 38.11 (C-4′), 45.97 (C-7′), 135.40 (C-2), 150.52 (C-3), 191.03 (-CHO).

(*E*)-*α*-*Santalal* (**5**). ^13^C-NMR (125 MHz, CDCl_3_) δ 9.05 (C-6), 10.54 (C-8′), 17.42 (C-9′), 19.66 (C-2′), 19.83 (C-6′), 24.44 (C-4), 27.45 (C-1′), 31.09 (C-5′), 31.55 (C-3′), 33.19 (C-5), 38.51 (C-4′), 46.15 (C-7′), 139.09 (C-2), 154.96 (C-3), 194.93 (-CHO).
