# Supplementary material for: Structure-Odor Relationships of α­Santalol Derivatives with Modified Side Chains
Source: Molecules. 2012 Feb 22;17(2):2259–70. doi: 10.3390/molecules17022259 (PMC6268821; doi:10.3390/molecules17022259)
Supplement: Supplementary File 1 [file molecules-17-02259-s001.pdf]

Molecules **2012**, *17*, 11292-11293; doi:10.3390/molecules170911292

OPEN ACCESS

molecules

ISSN 1420-3049

www.mdpi.com/journal/molecules

Correction

Hasegawa, T., *et al.* Structure-Odor Relationships of α-Santalol Derivatives with Modified Side Chains. *Molecules,* 2012, *17*, 2259-2270

Toshio Hasegawa [1,*], Hiroaki Izumi [1], Yuji Tajima [1] and Hideo Yamada [2]

[1] Department of Chemistry, Graduate School of Science and Engineering, Saitama University, Saitama 338-8570, Japan

[2] Yamada-matsu Co., Ltd., Kyoto 602-8014, Japan

* Author to whom correspondence should be addressed; E-Mail: toshihas@mail.saitama-u.ac.jp; Tel.: +81-48-858-3619; Fax: +81-48-858-3619.

Received: 23 July 2012; in revised form: 30 July 2012 / Accepted: 30 July 2012 / Published: 21 September 2012

The authors are sorry to report that some of the ^{13}C-NMR data reported in their recently published paper [1] were incorrect. While this manuscript was in preparation and pending recording of some ^{13}C-NMR spectra the data of α-santalol was used as a placeholder. Upon revising the manuscript according to the reviewers' comments, they mistakenly thought that all spectral data had been replaced by the correct measured data. Consequently the authors wish to make at this time the following corrections to the paper:

Section *3.2. Synthesis of a-Santalyl Benzyl Ethers and Separation of Their (Z)- and (E)-Isomers*

(*Z*)-*Benzyl ether.* ^{13}C-NMR (125 MHz, $CDCl_3$) δ 10.65 (C-8′), 17.47 (C-9′), 19.49 (C-2′), 19.51 (C-6′), 21.69 (C-6), 23.03 (C-4), 27.36 (C-1′), 31.00 (C-5′), 31.50 (C-3′), 34.83 (C-5), 38.13 (C-4′), 45.84 (C-7′), 68.39 (C-1), 71.68 (C-7″), 127.5 (C-4″), 127.7 (C-3″,5″), 128.3 (C-2″,6″), 130.6 (C-3), 131.4 (C-2), 138.6 (C-1″).

(*E*)-*Isomer of the benzyl ether.* ^{13}C-NMR (125 MHz, $CDCl_3$) δ 10.67 (C-8′), 13.80 (C-6), 17.51 (C-9′), 19.52 (C-2′), 19.56 (C-6′), 23.02 (C-4), 27.40 (C-1′), 31.02 (C-5′), 31.52 (C-3′), 34.18 (C-5), 38.19 (C-4′), 45.88 (C-7′), 71.47 (C-7″), 76.42 (C-1), 127.5 (C-3), 127.7 (C-4″), 128.3 (C-3″,5″), 129.2 (C-2″,6″), 131.7 (C-2), 138.7 (C-1″).

Section *3.5. Synthesis of α-Santalol Aldehyde Derivatives*

(*Z*)-*α-Santalal* (**4**). ^{13}C-NMR (125 MHz, $CDCl_3$) δ 10.51 (C-8′), 16.32 (C-6), 17.43 (C-9′), 19.35 (C-2′), 19.47 (C-6′), 22.26 (C-4), 27.31 (C-1′), 30.96 (C-5′), 31.45 (C-3′), 34.80 (C-5), 38.11 (C-4′), 45.97 (C-7′), 135.40 (C-2), 150.52 (C-3), 191.03 (-CHO).

(*E*)-*α-Santalal* (**5**). ^{13}C-NMR (125 MHz, $CDCl_3$) δ 9.05 (C-6), 10.54 (C-8′), 17.42 (C-9′), 19.66 (C-2′), 19.83 (C-6′), 24.44 (C-4), 27.45 (C-1′), 31.09 (C-5′), 31.55 (C-3′), 33.19 (C-5), 38.51 (C-4′), 46.15 (C-7′), 139.09 (C-2), 154.96 (C-3), 194.93 (-CHO).

Reference

1. Hasegawa, T.; Izumi, H.; Tajima, Y.; Yamada, H. Structure-Odor Relationships of α-Santalol Derivatives with Modified Side Chains. *Molecules* **2012**, *17*, 2259–2270.

© 2012 by the authors; licensee MDPI, Basel, Switzerland. This article is an open access article distributed under the terms and conditions of the Creative Commons Attribution license (http://creativecommons.org/licenses/by/3.0/).
